# A New Medical Journal in Boston

**Published:** 1842-04

**Authors:** 


*A New Medical Journal in Boston.*—It will be seen by an advertisement in to-day's Journal, that some preliminary efforts have been made with reference to the establishment of a quarterly Journal of Medical Science in this city, which, instead of exciting any alarm in regard to the effects it may produce on our own long cherished periodical, has our best wishes for its success. The gentlemen who are to be the editors, are competent to conduct its course with dignity and renown. Once or twice within the last few years, attempts have been made in this place to usher into existence a new medical periodical, but somehow they were smothered in embryo. The present enterprise, it is hoped, will succeed better. We never entertained an idea of monopoly in the way of Journalizing; the wonder is, how it happens that we have been, so many years, unmolested occupants of the field. Since there is room enough for more, and talent enough in this city and in New England to sustain half a dozen quarterlies, of the highest order, it only remains for the profession to contiuue their liberal patronage to the Boston Medical and Surgical Journal, and withal offer such pecuniary assistance as shall likewise place the contemplated quarterly on a firm foundation. Any facilities which we can offer through our own pages, towards forwarding the operations of the projectors, is cheerfully tendered to them.—*Boston Med. and Surg. Jour.*

